# A qualitative study of the current state of heart failure community care in Canada: what can we learn for the future?

**DOI:** 10.1186/s12913-015-0955-4

**Published:** 2015-07-28

**Authors:** Sean M. Hayes, Sophie Peloquin, Jonathan G. Howlett, Karen Harkness, Nadia Giannetti, Carol Rancourt, Nancy Ricard

**Affiliations:** AXDEV Group Inc., Brossard, QC Canada; Department of Cardiac Sciences and Libin Cardiovascular Institute, University of Calgary, Calgary, AB Canada; Hamilton Health Sciences, McMaster University, Hamilton, ON Canada; McGill University Health Centre, Montreal, QC Canada; Servier Canada, Laval, QC Canada

**Keywords:** Heart failure, Heart failure management, Chronic disease management, Health services, Qualitative study, Canada

## Abstract

**Background:**

In North America and other industrialized countries, heart failure (HF) has become a national public health priority. Studies indicate there is significant heterogeneity in approaches to treat and manage HF and suggest targeted changes in health care delivery are needed to reduce unnecessary health care utilization and to optimize patient outcomes. Most recent published studies have reported on the care of HF patients in tertiary care hospitals and the perspective of non-specialist stakeholders on HF management, such as general practitioners and clinics or hospital administrators is rarely considered. This study explores the current state of community-based HF care in Canada as experienced by various healthcare stakeholders providing or coordinating care to HF patients.

**Methods:**

This study employed a qualitative exploratory research design consisting of semi-structured telephone interviews conducted with health care providers and health care administrators working outside of tertiary care in the four most populous Canadian provinces. A modified thematic analysis process was used and the different data sources were triangulated. Findings were collectively interpreted by the authors.

**Results:**

Twenty-eight participants were recruited in the study: eight cardiologists, five general practitioners/family physicians, eight nurse practitioners/registered nurses, four hospital pharmacists and three health care administrators/directors. Participants reported a lack of stakeholder engagement throughout the continuum of care, which hinders the implementation of a coordinated approach to quality HF care. Four substantive themes emerged that indicated challenges and gaps in the optimal treatment and management of HF in community settings: 1) challenges in the risk assessment and early diagnosis of HF, 2) challenges in ensuring efficient and consistent transition from acute care setting to the community, 3) challenges of primary care providers to optimally treat and manage HF patients, and 4) challenges in promoting a holistic approach in HF management.

**Conclusions:**

As health systems evolve from tertiary-based care to community-based outpatient services for the management of chronic diseases, this study’s findings pinpoint challenges that have been observed in the Canadian context and can stimulate and orient dialogue toward solutions for a more coordinated approach to improve the care of HF patients and reduce pressure on the healthcare system.

## Background

Heart failure (HF) is a complex syndrome wherein a malfunction of the heart results in low cardiac output and pulmonary or systemic congestion contributing to recurrent hospitalizations, reduced quality of life, and death [1]. Heart failure patients often suffer from multiple co-morbidities that complicate optimal HF diagnosis and render patient self-management challenging [2, 3]. Despite recent advances in pharmacological and device therapy, and long established therapeutic guidelines for clinical practice, HF still has a very poor prognosis. For example, in Canada, the average lifespan of heart failure patients is 5.5 years [4], with a 1-year mortality rate of 25 % after diagnosis and a 30-day mortality rate of 16 % after hospitalization [5].

Approximately 1 in 4 HF patients is readmitted to the hospital within 30 days of discharge [6].

In North America and other industrialized countries, HF has become a national public health priority. For example, in 2009, in response to a call from the Government of Canada to develop a comprehensive national plan for heart disease, the Canadian Cardiovascular Society, the Canadian Heart and Stroke Foundation and the Canadian Institutes of Health Research proposed a Heart Health Strategy and Action Plan (CHHS-AP) [7]. Recognizing HF as a major cause of health care resource utilization, the Public Health Agency of Canada incorporated this action plan into its 2013–2016 Strategic Plan for chronic disease with the objective to reduce by 25 % the number of HF-related hospitalisations [8].

The recognition of HF as a Canadian public health priority led to the evolution of its model of care. The role of primary care to treat and manage HF was enhanced, shifting such care away from tertiary care institutions. Multi-disciplinary HF clinics were implemented; however the proliferation of these clinics occurred in the absence of a coordinated strategy and without specific guidance as to their structure [9, 10]. Studies suggest there is currently significant heterogeneity in HF care across and within health care regions [11, 12] with a wide spectrum of care models [13] and important disparities in referral with respect to gender, age, and type of HF [14].

The development of a more coordinated and standardized approach to HF care requires a better understanding of the current environment in which care is provided. Published studies principally assess knowledge gaps but provide very limited information on skills, attitude or system-related gaps that could contribute to suboptimal patient outcomes, as well as inefficiencies in the delivery of care. The perspective of non-specialist stakeholders on HF management, such as general practitioners and clinics or hospital administrators, is also very rarely considered. While most HF studies have reported on the care of HF patients in tertiary care hospitals, no published Canadian data are available on the current state of HF care in community settings.

This study was designed to provide an initial exploration of the current state of HF care in community settings in the Canadian context and to help identify the potential challenges and opportunities for improvement across the HF patient trajectory, as well as areas where further investigation would be required.

## Methods

**Fig. 1:**
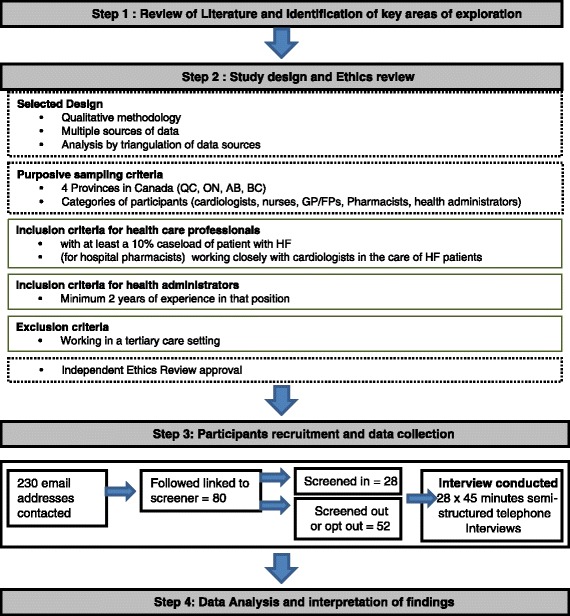
Details of the study design, recruitment and enrolment

The approach to this study is presented in Fig. 1 and included 4 steps: 1) literature review and identification of key areas of exploration, 2) study design and ethics review, 3) participant recruitment and data collection and 4) data analysis and interpretation of findings.

For step 1, a review of the literature was performed to identify the known gaps and challenges in HF care in Canada and to determine areas of exploration for this study. These areas of exploration (Table 1) served as a framework for designing the data collection tools.

**Table 1:** Selected areas of exploration, and examples of questions for the interviews

Areas of exploration in HF care in Canada		Examples of interview questions
Treatment	Current knowledge of the treatment options available	What are the current protocols in place in your practice/institution that helpdefine the choice of treatment and care of heart failure patients?
	Current protocol or algorithm followed for decision making around choice of treatment/medication	How are new therapeutic options integrated in practice as they are becoming available?
	Clinical decision making in end-of life (and consideration of patient expectations in clinical decision)	
Management	Current state of home care (and previsions/expectations for the future)	What changes are you expecting to occur in the next 5 to 10 years in relation to optimizing care in the Home care setting?
	Current state of hospital care (and previsions/expectations for the future)	Do you foresee any barriers for implementing those changes? What are the current challenges in the transition between the hospital and home care (transitional care)?
	Current state of transitional care (and previsions/expectations for the future)	
Collaboration	Roles and responsibilities of each Health care providers on multidisciplinary team	In your opinion, how optimal is the current sharing of roles and responsibilities between the members of the health care team who are involved in the careof HF patient?
	Role and responsibilities of the Health care providers shared with the caregiver	
Resource allocation	Current resources allocation throughout the system	Published literature refers to inappropriate resource allocation in Hospital and Community-based care/Home care as a reason for challenges experienced in Heart Failure care in Canada. What do you think about this statement?
	Current consequences of treatment choices being influenced by restrictive reimbursement	

For step 2, a qualitative exploratory research design was employed. Semi-structured Key Informant Interviews (KII) was the qualitative method selected because KIIs are valued means by which to explore the multiple subjective experiences intrinsic to the care and management of a disease, − particularly in a condition such as HF - wherein patients may have multiple interactions with different professionals and across different systems [15–18]. For recruitment in the study, invitations were sent by co-authors SH and SP to approximately 230 email addresses drawn from relevant email lists from each of the authors’ professional networks. A criterion purposive sampling strategy with maximum variation was used to enrol participants [19] because the aim was to obtain a diversity of opinions and perspectives. Categories of participants included cardiologists, family physicians/ general practitioners, nurse practitioners, hospital pharmacists, hospital administrators or health care network administrators. Inclusion criteria for health care providers (i.e., not including administrators) are detailed in Fig. 1 and included being actively practicing in a community-based setting, having at least five years of experience working with HF patients, and having a practice caseload of a minimum of 10 % HF patients. Administrators required to have been in a management position for at least 2 years. As HF care is for the most part provided outside of tertiary settings and limited studies reported on community based HF care, the authors opted to focus this study upon the reality facing community providers. Therefore, health care providers practicing within a tertiary care setting were excluded.

The study was submitted to an Institutional Research Board and received independent ethical approval (IRB Services, Aurora, ON). Research honoraria were offered to participants in respect of applicable ethical regulations [20].

For step 3, healthcare stakeholders were recruited by disseminating general information about the study in primary-care clinics and community-based hospitals. Recruitment was also done via an email invitation circulated in the author’s professional networks across the country. This recruitment method was judged sufficient to facilitate the purposive sampling required for this exploratory study. The email included a link for potential participants to confidentially complete a screening questionnaire online. Eligible participants also provided online their availabilities for a telephone interview. Recruitment was stopped after each potential participant on the list had received 2 reminders.

Opinions of health care stakeholders regarding the current state of HF care in their regions were collected through semi-structured telephone interviews. The interviews were conducted by trained qualitative interviewers in the participants’ language of choice (either English or French). Interviews lasted approximately forty-five minutes and were audio-recorded. Examples of questions included in the interview guide are included in Table 1. Questions were developed in a multidisciplinary fashion, preventing undue influence by one researcher. Informed consent was obtained through an online opt-in that also included information on how anonymity and confidentiality would be ensured, and questions to ensure eligibility. The interviews focused on the challenges experienced in caring for HF patients and the future changes perceived as needed to further improve the quality of HF care. The interviewers’ notes and interview recordings were used to debrief SP and SH who performed the initial analysis and aggregated the data. All authors but SP were blinded to the participants’ names and identifying information.

For step 4, a modified thematic analysis approach was used to analyze the data [21]. This approach involved three steps: data familiarization (where the researcher immerses him/herself in the data), data coding (codification and classification of data according to areas of exploration), and theme identification (where themes with substantial data emerge from the coding). This inductive approach was deemed appropriate considering the exploratory and descriptive objective of the study. Source triangulation was used to identify the important themes reported by more than one category of stakeholders and in more than one province. As the aim of the study was to identify main trends in the current state of HF care in community settings, less attention was given to negative or deviant cases. All authors were involved in a multi-disciplinary interpretation of blinded data to determine the grouping of the themes under larger categories of challenges reported to impact the provision of optimal HF care in community-based settings. The multi-disciplinary aspect of the interpretation process ensured that each researcher‘s perspectives and opinions were integrated, mitigating the risk of undue influence of one researcher on the overall conclusions of the study. Furthermore, this also allowed for discussion of potential alternative causalities for the findings than those reported by interviewees. Disagreements were resolved through discussion, and revisiting the data collected if needed.

## Results

### Sample

Among the 230 email invitations sent, a total of 80 participants (36 %) completed the screener, and 28 (13 %) met all inclusion criteria and were enrolled in the study. The most common reasons for exclusion were practice within a tertiary care setting and less than 10 % of their patient caseload with HF. Our cohort included eight cardiologists, five general practitioners, eight nurse practitioners/registered nurses, four hospital pharmacists and three health care administrators/ directors. Participants’ characteristics are presented in Table 2. Most participants were experienced health care providers (82 % with over 10 years of experience), with HF patients representing between 10 and 50 % of their patient caseload.

**Table 2:** Characteristics of participants (n (%))

Years of practice (*n* = 28)	
2–5	2 (7 %)
6–10	3 (11 %)
11–20	10 (36 %)
21–30	7 (25 %)
>30	6 (21 %)
Practice Setting (*n* = 25)	
Urban	18 (69 %)
Suburban	7 (31 %)
Rural	0 (0 %)
Caseload HF (*n* = 20)	
10–30	9 (45 %)
31–50	7 (35 %)
51–75	1 (5 %)
>75	3 (15 %)
Gender (*n* = 28)	
Male	15 (54 %)
Female	13 (46 %)
Province (*n* = 28)	
Alberta	5 (18 %)
British Colombia	5 (18 %)
Ontario	12 (43 %)
Quebec	6 (21 %)
Profession (*n* = 28)	
Cardiologists	8 (29 %)
General Practitioners	5 (18 %)
Nurses/Nurse Practitioners	8 (29 %)
Hospital Pharmacists	4 (14 %)
Healthcare Administrators/Directors	3 (11 %)

### Challenges to optimal heart failure care

**Fig. 2:**
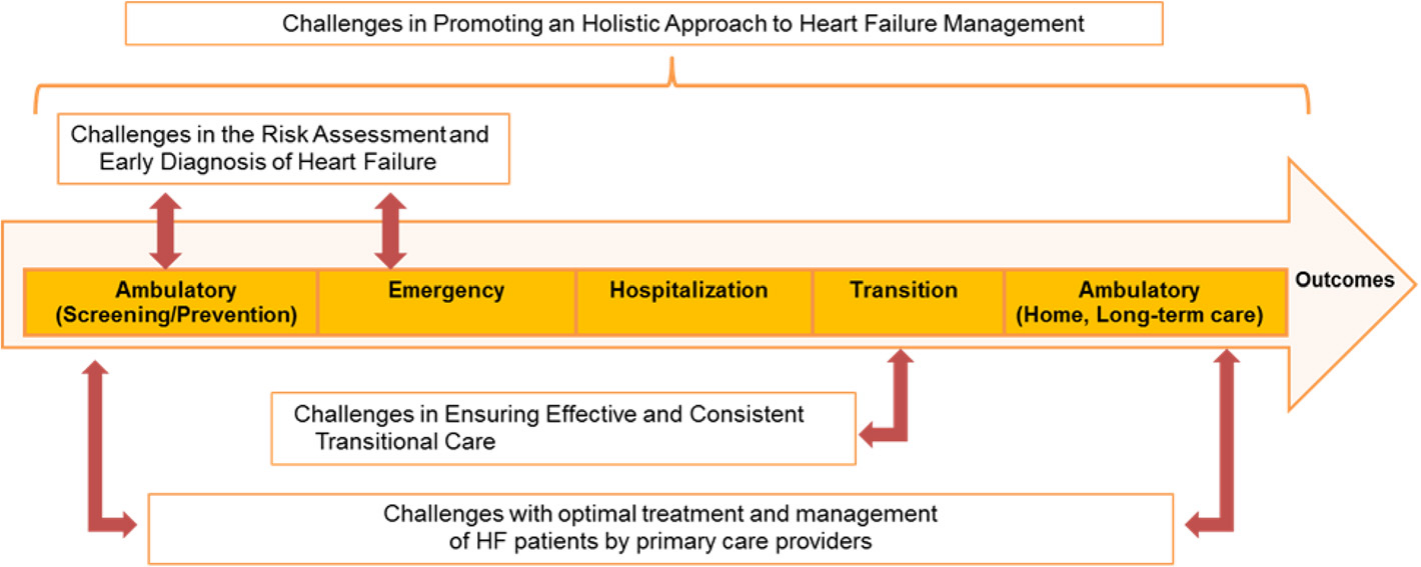
Four key emerging themes challenging heart failure care in Canada

Several gaps and barriers challenging optimal care were identified from the data. Those have been regrouped into four main themes representing substantive challenges for the provision of optimal HF care across the continuum of care: 1) challenges in the risk assessment and early diagnosis of HF, 2) challenges to ensure an efficient and consistent transition from acute care setting to the community, 3) challenges of primary care providers to optimally treat and manage HF patients, and 4) challenges in promoting an holistic approach to HF management (see Fig. 2). In the following sections, each key theme is detailed with their causal factors as reported by participants.

#### Challenges in the risk assessment and early diagnosis of heart failure

Early diagnosis of HF is rendered possible only if health care professionals are knowledgeable regarding what actually constitutes HF and how to diagnose it. This was reported as a challenge by participants.

“*Part of the problem has to be: recognize it’s HF to begin with. It’s too easy for Family Physicians and maybe Emergency Physicians to label patients with HF*”. - Administrator, Ontario.

Participants reported that proactive risk assessment and early diagnosis of HF is hampered in the presence of multiple co-morbidities among this patient profile. As one cardiologist participant from Ontario remarked:

*“When it comes to etiology, it is not always easy to differentiate. […] There is no one test that says : you have heart failure”.*

Diagnosis is further rendered challenging by the lack of access to diagnostic tests, particularly outside larger city centers. In some settings, primary health care providers reported having little access to cardiologists for consultation regarding complex cases.

*“It’s difficult even for us as health professionals, we would need support from cardiologists or specialised centers. (…) In my region, there is no cardiologist, zero, so I have to refer in other regions, which is difficult. Access to cardiologist, but also access to diagnostic tests is difficult. Sometimes we would like to have an expert opinion but we can’t have it”.* -Nurse Practitioner, Quebec (translated from French).

*“There is ways of getting around the barricades for getting diagnostic testing because we are in the lower mainland, but if you were up north, to get an ultrasound test, you can wait months. So the equity of testing is very variable depending on where you live”.* - Registered Nurse, British Columbia

#### Challenges in ensuring effective and consistent transitional care

Apart from a few large hospital networks, protocols for HF are rarely reported to be implemented in participants’ practice settings. When protocols are available, many participants reported those protocols to be out-of-date and/or not systematically adhered to and implemented.

*“Not everybody that is admitted with HF would be put on the protocol; I think that could be improved. I don’t think everybody is aware that the protocols exist. There is just so many in place. Often times the admitted HF patients are complex. […] HF is just one of the several admitting diagnosis”* - Cardiologist, British Columbia

Effective and consistent transitional care from the inpatient setting and/or the Emergency Room to outpatient care is perceived as essential to optimal HF care by all participants. However, participants reported challenges in effecting a smooth and optimal transition for both patients and for the treating professionals. The underlying causes for these challenges included limited resources, lack of opportunities for proper patient education by Nurses, Pharmacists or other health care staff, and a lack of systematized processes and channels for proper transfer of information among and between treating physicians. Early discharge and improper transition were perceived by participants as contributors to the high national readmission rates.

*“We have some capacity challenges in terms of transition of care. In the case of heart failure, when a patient gets admitted to the ER, [what we need is] having a discharge plan that links to a provider follow-up. Any solutions that we could have to bridge that challenge, that would be good”.* - Administrator, Ontario.

*“In Alberta, there is a real push for early discharge […] appropriate resources are not always set up for the patient before discharge”.* - Registered Nurse, Alberta.

*“There is no transitional care at all. They just go […] They don’t get any counselling from the pharmacist in the hospital […].We don’t have any education component for HF […] I can’t even tell you they get a pamphlet”.* – Hospital Pharmacist, British-Columbia.

In this context, health care providers in hospital settings rely heavily on patients to relay the information to other community based health care providers and be their own health advocate. Given the advanced age and overall health condition of these patients, this raises concerns for many providers regarding continuity of care and safety issues.

*“We send them out, we tell the patient very clearly that their ACE-inhibitor or beta blockers are not blood pressure pills and for no reason, if your blood pressure is low, not let anybody stop it”.* - Nurse practitioner, Ontario.

HF clinics are understood by participants as the appropriate and optimal setting to provide education to patients about their disease, and are acknowledged for their effective, smooth and successful transition of patients. However, participants who did have knowledge of HF clinics in their area reported such clinics are generally overbooked and understaffed. Access to HF clinics is further complicated for many patients due to transportation issues. Other participants acknowledged that they were not well informed about the services provided by HF Clinics in their regions.

#### Challenges of primary care providers to optimally treat and manage HF patients

Participants reported that General Practitioners and Family Physicians (GPs/FPs) are lacking adequate knowledge in recent advances and evidence-based treatments for HF; and lacking the skills required for the optimal management of HF. Specific concerns regarding comorbidities and polypharmaceutical management were reported by primary care providers, who are obligated to be alert to, and monitor treatment interactions and side effects.

*“If primary care physicians were taught and educated and encouraged to seek these things out (signs of fluid retention) we may be able to do a better job at avoiding hospital visit with frank failure”.* - General practitioner, Ontario.

*“I would like to know that I am doing the best that I can for those patients, but I feel that is I am just given them a diuretic, a beta-blocker and an ACE-inhibitor, and I check their blood work once in a while, I feel this is the limit of what I know how to do, and I would like to know what the next steps could be”.* - General Practitioner, British Columbia.

Even with the proper knowledge and skills, it was recognized by GPs/FPs and other stakeholders alike, that primary care providers lack the time and support needed to ensure efficient and effective follow-ups and accurate monitoring of HF patients.

*“Continuing an ACE-inhibitor is on the guidelines for HF treatment, but they (family physicians) may feel really uneasy if their renal function start to decline and then they will stop it right away”.* - Nurse Practitioner, Ontario.

#### Challenges in promoting an holistic approach to heart failure management

*“Heart failure is still treated by primary care physicians as an acute illness. […] Therefore, we don’t see our patients in the lens of monitoring HF to prevent exacerbation, rather we react when they have exacerbation”.* - General Practitioner, Ontario.

Participants in all stakeholder categories reported the importance and value for HF to be treated and managed as a chronic condition, requiring a coordinated multidisciplinary approach. Participants emphasized the active role that community-based health care providers, including GPs/FPs, nurses, nurse practitioners and community pharmacists, should play in HF care.

*“The community pharmacist need to be educated on the disease of HF and what to counsel patients, on the importance of medication and what symptoms to look for and things that can trigger it. I don’t know if community pharmacists are checking: have you gain weight, when have you done your last blood test?” *- Hospital Pharmacist, British-Columbia

*“I really think that the cardiologist have to press upon the primary care physicians saying “Listen you can prevent emergency room visits in heart failure […] by just taking a little extra time to check over your patients. Just like we do for Diabetes […] the heart failure patient should have dedicated visit specifically for heart failure”. -* General Practitioner, Ontario.

The paradigm shift from episodic diabetes treatment and management to a chronic care approach was alluded to as an example for HF. Participants acknowledged this would require a shift in roles and responsibilities of all stakeholders, including the patients. For example, health care providers would require to be trained to promote HF patient proactive self-management.

*“The training of professionals to practice that way, they are not taught that. We need to look at how we are training our physicians. […] Some of these chronic disease models need to be given more high profile in the curriculum, including self-management”* - Administrator, Ontario

## Discussion

Heart failure has been singled out as an emerging epidemic [22–24]. Of the major forms of cardiovascular disease, epidemiological studies have shown HF to be the only disease that is increasing in incidence and prevalence in most developed countries, affecting nearly 5.8 million people in the United States, and over 23 million worldwide [23]. Other authors however have stated that over the last decade, the incidence of HF remained overall stable, while survival improved and as a result, the prevalence of HF has increased, creating an epidemic which is mostly one of hospitalizations [24]. These authors state that the increasing number of cases could be explained by changes over the years in payment systems, in how discharge information is reported, and in the use of validated standardized criteria, without an actual increase in incidence [24]. In the context of an aging population and a pressured healthcare system trying to reduce hospitalization rates, the multi-disciplinary nature of HF care calls for a well-coordinated and seamless patient management approach along the whole continuum of care. Unfortunately, very limited information is available to describe how our healthcare system is currently performing.

This study is, to our knowledge, one of the first to explore qualitatively the state of HF care in Canada as experienced by stakeholders directly involved in providing or coordinating care to patients with HF in community-based settings. Our premise and design was qualitative in order to explore and generate hypotheses, and the findings suggest that four primary challenges are to be addressed to improve the continuum of HF care. Identifying opportunities for improvement along the continuum of HF care could contribute to reducing hospitalization and re-hospitalization rates of HF patients [25–27].

One means by which this could be achieved is by ensuring a proper and early diagnosis of HF in the community setting, especially by non-cardiologist specialists [28]. This would require that primary care providers be given opportunities to increase their knowledge, skills and confidence in the accurate identification of patients at risk of HF or in the diagnosis of HF. Similarly to what was found in this study, a reported lack of confidence in establishing an accurate diagnosis of HF was reported among General practitioners in the UK. The potential causes mentioned for this lack of confidence were: perception of a high risk of mis-diagnosis among GPs and the lack of access to diagnostic tools by GPs [17].

Other Canadian studies have reported on the lack of efficient and systematic transitional care from the hospital setting back to the community setting. In the Canadian province of Quebec, among 401 HF patients discharged after a visit to the emergency department, only half of the patients (51 %) were seen by a physician within 4 weeks after discharge as recommended by clinical practice guidelines [29, 30]. In the provinces of Alberta and Ontario, 34 % and 19 % of patients, respectively, had no physician follow-up within 30 days after discharge [31, 32]. Although there is a process for transitional care in approximately 80 to 90 % of hospitals in Ontario, this practice is not standardized [33]. Many underlying causes have been identified in relation to challenges in transitional care, including the lack of standardized interventions; the lack of risk stratification to prevent overuse of resources; and the lack of cost analyses related to interventions being put in place [34]. Making effective transitional care a measured performance indicator could incent health care institutions to take clear steps towards ensuring optimal shift of care to community based health care providers.

Effective transitional care is dependent on the skills and the availabilities of health care professionals to ensure the transition to the community settings. Currently, the number of practicing cardiologists remains lower than the demand for consult [17]. The delay for cardiologists to see non-urgent cases in Canada has been reported to be more than three months, enough for the non-urgent cases to ultimately exacerbate and re-enter the system through the emergency department [35]. In a study on the type of physicians treating acute cardiac conditions, Tu et al. reported GPs/FPs are responsible for the treatment and management of half the population of hospitalized HF patients in Canada [12]. Similar findings have been reported in Alberta and Quebec where half of HF patients had been diagnosed by a primary care physician in outpatient settings [36, 37]. However, patients diagnosed by a GP have been reported to have a longer wait until their first consultation with a cardiologist [36].

This calls for a shared responsibility between the specialists and primary care providers who need to be more engaged and accessible for ongoing HF patient monitoring. Better outcomes have been observed when HF patients are treated by both a GP and a specialist [32, 33]. However, studies have reported on GPs’ uncertainty about their capacity to properly initiate treatment using ACE-inhibitors and Beta-blockers, weighting the risk and benefits of each treatment and managing the associated symptoms and side effects [16, 17]. This study confirmed primary care providers’ challenges in treatment and management of HF patients in community-based settings, thereby suggesting a need for targeted HF education, tools and support directed to the primary care provider.

Part of the solution also lies in the implementation of specialized community based HF clinics, which can be nurses-led and provide multi-disciplinary care to patients in the community [38]. Specialized multidisciplinary community-based care has been reported to reduce mortality in HF patients by 29–40 %, reduce all cause hospitalization by 12 %, and reduce HF specific hospitalization by 25–27 % [39–41]. However, most of these clinics are operational in large health care institutions and are poorly represented in small or community hospitals. Thus, only a minority of patients with HF have access to HF care through a dedicated clinic. In Ontario, 8.9 % of patients hospitalized for HF will be seen in one of the province HF clinics within 1 year [42]. In Quebec, HF patients will wait on average 1.2 years from time of diagnosis before getting admitted to a HF clinic [43].

Could the expertise developed by HF clinics be shared with primary care providers so they feel more confident in their ability to provide routine care and basic disease management to HF patients in their own clinical settings, and therefore broaden the reach to patients in community settings? This could not only alleviate specialized HF services to have the capacity to see more referrals, faster, and to provide care plans for HF patients, but also allow them to focus on complex and difficult cases that can less easily be handled by primary care physicians.

The Canadian health care stakeholders that participated in this study acknowledged the importance of adopting a holistic and integrated approach to care in HF, and the benefits that could be gained by recognizing HF as a chronic rather than acute condition. The Chronic Care Model, developed by Edward H. Wagner and colleagues [44], suggests to revisit the way care is provided in chronic disease by redesigning the delivery system in ways that promote collaboration and transferring the bulk of the management of the chronic conditions to non-specialized health care providers, promoting better use of community resources with the aim to further empower the patients, and ensuring evidence-based best practices are integrated into daily practice for all health care providers involved [45, 46]. Interventions implemented in light of this model have been reported successful in chronic disease including diabetes, asthma and HF [47, 48]. In HF, these interventions were indicated to be beneficial to improve continuity of care, as well as efficiency and cost-effectiveness of care [48].

For the purpose of this exploratory study with a small sample size, we used a purposive sampling to ensure the participants interviewed were experienced in HF (at least five years of experience in the field, with HF patient representing more than 10 % of their caseload), were working outside the specialized environment (tertiary care hospital), and were coming from one of the four largest provinces in Canada. Invitation to participate in the study was done by emailing to potential participants in the authors’ networks. For this reason, the opinions and perceptions of this study sample may not represent the opinions and perceptions of all practicing healthcare providers or stakeholders in the field of Heart Failure in Canada. The recruitment strategy, the small number of participants in each stakeholder categories and the fact that some provinces were not represented, implies limitations regarding generalizability of the findings. Finally, given the Canadian Health care systems are provincially run and publicly funded, it is also expected that those findings may not reflect the care provided under other funding models. It would be of value to conduct similar exploratory studies in other countries where a publicly funded health care system is in place, to assess if the issues uncovered throughout the trajectory of HF care in this study can be observed elsewhere.

Given that some of the challenges and recommendations discussed in this paper involved the contribution of HF patients and their caregivers, it would have been important to gather their perspectives regarding the state of HF care. The initial intention of co-authors at the launch of this project was to also include patients and caregivers. However, this population, given their advanced age and generally limited use of internet and technologies is challenging to recruit, especially if looking to recruit patients that are not benefiting from best standard of care through HF clinics. There is also a lack of official patient advocacy group for this specific population nationally which render their recruitment possible mainly via practicing health care providers in community settings. This could be the aim of future studies.

## Conclusions

HF is a chronic condition affecting mainly an older and medically complex population incurring burden on the patient, the family, healthcare providers, and the systems in which they engage. This study gathered the perspectives of health care stakeholders with extensive and relevant experience in HF. These professionals have called for a more systematic and coordinated approach to care in HF as part of the solution to promote better self-management of HF patients, prevent unnecessary or preventable hospitalization, and reduce the economic burden of HF. Achieving this coordinated care would require specific organizational changes to better support providers, especially in primary care, so they can feel better equipped to care for this patient population. In the Canadian context, as the provincial health systems evolve from tertiary-based care to community-based outpatient services for the management of chronic disease, this study assessing the current state of care throughout the HF trajectory is a step forward finding solutions to optimize HF care and render HF treatment and management more proactive and preventative. Given the burden that represents HF globally, this study could be useful to decision makers in other legislations to orient discussions on the current situation of HF care in their own regions.
